# Environmental Chemical Contaminants in Food: Review of a Global Problem

**DOI:** 10.1155/2019/2345283

**Published:** 2019-01-01

**Authors:** Lesa A. Thompson, Wageh S. Darwish

**Affiliations:** ^1^Veterinary Teaching Hospital, Faculty of Veterinary Medicine, Hokkaido University, Sapporo 060-0819, Japan; ^2^Laboratory of Advanced Lipid Analysis, Department of Health Sciences and Technology, Faculty of Health Sciences, Hokkaido University, Sapporo 060-0812, Japan; ^3^Food Control Department, Faculty of Veterinary Medicine, Zagazig University, Zagazig 44519, Egypt

## Abstract

Contamination by chemicals from the environment is a major global food safety issue, posing a serious threat to human health. These chemicals belong to many groups, including metals/metalloids, polycyclic aromatic hydrocarbons (PAHs), persistent organic pollutants (POPs), perfluorinated compounds (PFCs), pharmaceutical and personal care products (PPCPs), radioactive elements, electronic waste, plastics, and nanoparticles. Some of these occur naturally in the environment, whilst others are produced from anthropogenic sources. They may contaminate our food—crops, livestock, and seafood—and drinking water and exert adverse effects on our health. It is important to perform assessments of the associated potential risks. Monitoring contamination levels, enactment of control measures including remediation, and consideration of sociopolitical implications are vital to provide safer food globally.

## 1. Introduction

**Figure 1:**
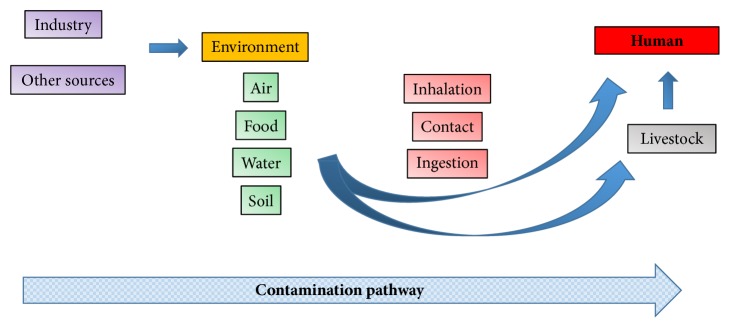
Sources of environmental contaminants in human foods.

Chemical contamination is a global food safety issue. There are many potentially toxic substances in the environment which may contaminate foods consumed by people. They include inorganic and organic substances and may originate from a wide range of sources (Figure 1 shows the pathway of contaminants through the environment). This review is restricted to chemical contamination of foods and does not address biological or physical hazards.

In certain instances, the source of contaminants may be the environment. This is the case for metals such as lead and mercury, dioxins, and polychlorinated biphenyls (PCBs). Agricultural use of pesticides may lead to food contamination. Similarly, drugs used in both people and animals may contaminate waterways and pose a health risk to consumers. Additionally, food packaging methods may be a source of contamination, so-called “migrants” leaching from packing materials. These contaminants may cause acute or chronic toxic effects. Toxicity may relate to the route of exposure and dose, and personal characteristics such as age and health condition may affect the individual's susceptibility.

Due to the nature of contamination, some food products may be more contaminated than others. This may be due to several factors such as varying exposure to pesticides, differences in plant uptake mechanisms from the environment, or contaminants from food packaging [1, 2]. Dietary make-up will affect an individual's exposure to these contaminants. For example, nursing neonates have a high intake of contaminants that are excreted in breast milk [3]. Exposure at different life stages may result in different toxic effects as well. For example, prenatal exposure to persistent organic pollutants has been linked to an increase in childhood obesity and increased blood pressure [4].

For many food items—including vegetables, fish, and other seafood—human health risk assessment data is available after analysis of available foods [5–7]. Urban farms and gardens may pose additional risks due to contaminants such as metals [8, 9]. Furthermore, drinking water may become contaminated [10, 11]. Xenoestrogenic compounds have even been detected in rainwater [12]. Water contamination may also result in pollution of marine biota, affecting suitability for consumption of seafood [13]. Consequently individuals with high consumption of seafood will intake higher levels of such contaminants. Occupational exposure will not be discussed in detail in this review, but workers may have increased risk of exposure to certain contaminants, for example, car repair workers with lead on their hands which they ingest after hand-to-mouth contact [14].

For most contaminants, there is no completely safe dose level. However, for many, acceptable levels have been calculated—levels below which signs of toxicity should not be evident. Toxic effects seen depend on the contaminant in question, the dose received, and the individual. For example, many contaminants have been linked to an increased risk of cancer. Skin cancer has been associated with long-term exposure to drinking water contaminated by arsenic, gastric cancer with lead contamination, and liver cancer with consumption of grain contaminated by mercury [15–17]. Our understanding of the health risks from combined exposures to more than one contaminant and the means by which we can assess such interactions is lacking [18].

Monitoring programmes are in place both nationally and globally to monitor such contamination in order to assess food safety. However, it is important to note that such monitoring cannot completely preclude supply of contaminated food to consumers. The role of such programmes is to check that food and water contamination levels are below those deemed “unsafe.” To this end, many governmental and nongovernmental organizations strive through risk assessments to ascertain what levels of contamination are acceptable for products destined for human consumption. In addition, national and international policies are in place to reduce contamination. For example, under the Stockholm Convention on Persistent Organic Pollutants, production and use of such substances are eliminated or restricted. This international treaty came into force in 2004 and currently has 152 signatories from 182 parties [19]. The Codex Alimentarius describes international food standards, setting permitted maximum levels (ML) for contaminants in foods based on risk assessment and scientific evidence [20]. The Codex Committee on Contaminants in Food (CCCF) is a global forum, but it can be difficult to compromise national legislations and harmonize global standards. Lists of contaminants also undergo risk assessment by the Joint FAO/WHO Expert Committee on Food Additives [21, 22]. Recommendations are made for standards such as provisional maximum tolerable daily intake (PMTDI) or provisional tolerable weekly intake (PTWI). These are usually calculated based on chronic toxicity data, and thus it may also be useful to consider acute reference doses (ARfDs). The Codex Committee on Pesticide Residues (CCPR) has established maximum residue limits (MRLs) for over 5,000 pesticide residues [23]. This committee also considers reports from the FAO/WHO Meeting on Pesticide Residues (JMPR), which estimates MRLs and acceptable daily intakes (ADIs) for people [24].

## 2. Sources of Contaminants from the Environment to Food and Water

It is useful to consider the sources of contaminants in order to understand their pathway into food and water sources for consumption. Factors such as soil properties, activities by people, and point sources affect the accumulation of metals in the environment. For example, mining may result in release of substances such as arsenic and mercury [25, 26]. Once in the environment, these substances may contaminate food and water and result in human health hazards, with toxic effects varying depending on the contaminant(s) ingested (Table 1).

### 2.1. Metals and Metalloids

Metals and metalloids in the environment have various sources. One source of mercury and lead is artisanal gold mining. For example, in the gold mining area of Tongguan, Shaanxi, China, concentrations of these metals in locally produced grains and vegetables exceeded governmental tolerance limits and posed a potential health risk to people from consumption [58]. Lead and cadmium from an iron mine in Morocco resulted in concentrations of cadmium in livestock organs higher than acceptable limits [59]. Likewise, in Spain, sheep near a mine were found to have lead contamination, with levels in 87.5% liver samples above European Union Maximum Residue Levels (MRL) [60].

Industrial regions often have extensive environmental contamination by metals. In Romania, lead, cadmium, copper, and zinc contaminated crops, exceeding maximum acceptable levels in some samples [61]. In China, cadmium from a zinc smelter contaminated leaf and root vegetables particularly [62]. Arsenic, selenium, lead, and other metal and metalloid contaminants were found near a coking plant in China, contaminating soil and food, and detected in blood samples from children [63]. In that case, ingestion of food was determined to be the major exposure pathway for local children. In Belgium, cadmium was detected in locally produced food items grown near nonferrous metal plants [64]. Thallium from a steel plant in south China was found to contaminate soil and thence vegetables, exceeding German standards for the maximum permissible level and showing hyperaccumulation in plants such as leaf lettuce, chard, and pak choi [65].

Many fruits and vegetables have been shown to be contaminated by metals. For example, cadmium in soil was detected in navel oranges in China and lead and cadmium in soybeans in Argentina [66, 67]. Also in China, various metals were detected in edible seeds, with levels of copper sufficiently high to show an increased health risk to people consuming them [68]. On the contrary, contamination levels of mercury in rice samples from a city in eastern China were below levels likely to affect human health [69]. In the global arena, methylmercury has been detected in fish and other seafood around the world [70, 71]. Fish tissues from Turkey were shown to be contaminated with copper, iron, zinc, and manganese [72]. Various metals have also been detected in fish from Sicily, with some concentrations exceeding European regulation limits [73]. In Asia, food species of turtles have been shown to contain mercury [74].

With regard to water, endemic arsenism from contaminated drinking water has been reported in China [11]. Monitoring has detected nickel in drinking water in New South Wales, Australia, but levels do not appear to pose any health risk for the local population [75].

Further evidence of potential health risks to people from metals are surveys of human samples. Mercury and monomethylmercury were detected in human hair samples from French Guiana, associated with a diet rich in fish, with 57% of people tested having mercury levels higher than the WHO safety limit [70]. In Spain, mercury, lead, and cadmium have also been detected in human milk samples, with increased levels of lead associated with higher consumption of potatoes [76].

### 2.2. Polycyclic Aromatic Hydrocarbons

Polycyclic aromatic hydrocarbons (PAHs) primarily occur after organic matter undergoes incomplete combustion or pyrolysis, or from industrial processes [77]. Food contamination comes from the environment, industry, or home cooking (such as when using biomass fuels). These compounds appear to be genotoxic and carcinogenic. Oil spills from transporter ships in the ocean are all too common and will result in contamination of seafood. Besides the petroleum-related polycyclic aromatic hydrocarbon (PAH) compounds, chemical dispersants are often used to mitigate effects of oil in the ocean. After the BP Deepwater Horizon oil spill in Louisiana, USA, in 2010, the Federal government responded to seafood safety concerns by instigating protocols for sampling and analysis of food to determine its safety [78]. Lessons learned after this scenario included recognition of the need to improve risk assessments to adequately protect vulnerable populations, including pregnant women [79].

### 2.3. Industrial Chemicals

Persistent organic pollutants (POPs) are synthetic organic chemicals; some are used in industry, some as pesticides, and some are by-products from industry or combustion. They include pesticides like aldrin, chlordane and DDT, industrial chemicals like PCBs and HCBs, and unintended by-products like dibenzodioxins and dibenzofurans. They persist in the environment, are distributed globally in air and ocean currents, and accumulate in animals in the food chain (including in humans). Their side effects depend on the chemical and the contaminated species; for example, they may have effects on reproductive or immune systems, or increase cancer risks [80].

Chlorpyrifos is an organophosphate pesticide that affects vision and causes other neurological toxic effects in humans [81]. It has been detected in dietary samples, and foods have been shown to be responsible for approximately 13% of daily exposure to this chemical [51]. Organochlorine pesticides such as DDT have been used in agriculture and vector-transmitted disease control for decades, though their use now is restricted due to known persistence in the environment and toxic effects such as neurological dysfunction and endocrine disruption [82]. Pyrethroids such as permethrin and deltamethrin are widely used for control of vector insects and aircraft disinfection, as they are relatively safe for people [83]. However, their use near foods can result in contamination and studies are ongoing to reduce potential toxic effects [84, 85]. Although neonicotinoids are widespread in the environment and contaminate consumable items, their toxic effects are still not yet well understood [86, 87].

Polychlorinated biphenyls (PCBs) have a variety of uses in industry, including in transformers, as heat exchange fluids or paint additives, or in plastics. Ingestion of PCB residue-contaminated food—especially meat, fish, and poultry—is the main source for people, with ready absorption from the gastrointestinal tract [88, 89]. Contaminated breast milk is a potential source for nursing infants. Chloracne is reported after extensive exposure to PCBs, but immune and carcinogenic effects may also result.

Polybrominated and polychlorinated compounds may originate from anthropogenic and natural sources. They have many uses such as flame retardants and dielectric/coolant fluids in electrical apparatus. Toxic effects include endocrine disruption, neurotoxicity, and cancer. Polybrominated diphenyl ethers (PBDEs) and polychlorinated biphenyls (PCBs) have been detected in human milk in China [90]. This is a particular concern due to the high susceptibility of nursing infants to toxic effects. According to a study in Germany, dietary exposure is the most significant pathway for PBDEs in people [91]. In particular, seafood has been cited as a major contributor [92].

Perfluorinated compounds (PFCs) are synthetic chemicals with friction-resistant properties that make them useful in many materials and industries. Toxic effects include endocrine and immune system disruption and developmental problems. Some precursors or metabolic intermediates for perfluoroalkyl and polyfluoroalkyl substances (PFASs) are toxic, for example, estrogen-like activities [93]. The PFAS group includes perfluorooctane sulfonic acid (PFOS) and perfluorooctanoic acid (PFOA); these have been detected in many food sources including seafood in China and Germany [94, 95]. Drinking water and food are the main sources of exposure to PFOS and PFOA, although levels are usually low [96].

Acrylamide occurs in many foods—generally associated with high heat cooking processes (e.g., in breads and baked or fried potatoes)—and is also manufactured for commercial and industrial uses (such as in paper and dye production, in wastewater treatment, and as a chemical grouting agent) [97]. The IARC has classified acrylamide as a probable human carcinogen, placed in group 2A since 1994 [98].

### 2.4. Pharmaceuticals and Personal Care Products

The term pharmaceuticals and personal care products (PPCPs) includes a wide range of substances that may enter the environment and thence food or water sources. Antimicrobials and other drugs may originate from use in both humans and animals. For example, swine waste containing antimicrobials may contaminate both water and food [99]. Aside from the very real threat of increased antimicrobial resistance through exposure to extraneous sources of these chemicals, it has also been shown that many drugs have other side effects including endocrine disruption [100]. In some circumstances, the medicinal products themselves may be contaminated, for example, in many herbal products [101].

### 2.5. Radioactive Elements

Most radioactive elements did not exist naturally, and soil contamination with such material has only become a problem since nuclear weapons and reactors have been developed [102].

After tsunami damage affected the Fukushima nuclear plant in Japan in 2011, monitoring of food and water samples detected contamination above provisional regulation values and restrictions were put in place [103]. Radionucleotides have also been detected in seafood in India, various foods in the Balkans, and food and drinking water in Switzerland [104–106]. Risk assessments are conducted to ensure that levels remain within acceptable limits. Furthermore, experimental models are undertaken to assess safety in ingestion pathways, considering several different food intakes [107]. In the US, there is an FDA rule pertaining to uranium, radium, alpha particle, beta particle, and photon radioactivity in bottled water [108].

### 2.6. Electronic Waste

Modern society has become encumbered with many electrical devices, and electronic waste (or e-waste) has become a major problem. Inappropriate processing, for example, incomplete combustion, of such products releases a variety of pollutants covered above, including PBDEs, dioxins/furans (PCDD/Fs), PAHs, PCBs, and metals/metalloids [109]. In addition, contamination from such devices can enter drinking water and food [110].

### 2.7. Plastics

In recent times, we rely more and more on packaging materials—in particular plastics—to transport and help preserve food. These materials are not inert and may themselves contaminate food and drinks as multiple chemicals are released into foods and beverages from food contact materials. These are termed “migrants” and include such chemicals as phthalate plasticizers which have been detected in bottled water [111]. Factors such as higher storage temperatures and prolonged contact time with the packaging were linked to higher levels of contamination, but a health risk assessment showed that the risk for consumers was low [111].

### 2.8. Nanoparticles

Another recent development is that of nanoparticles. These have one dimension less than 1 x 10^−7^ m, and engineered nanoparticles have been used in a wide range of products, such as paints, cosmetics, and pesticides [102]. Pathways and effects of these in biota are as yet unclear, but they have been shown to travel in the food chain [112]. Nanosized materials have been detected in foods such as wheat-based products [113].

## 3. Risk Assessment and Monitoring

As shown in Table 1, each of the possible contaminants in food can be linked to a variety of toxic effects. Any adverse effects seen depend on multiple factors, including whether exposure is acute or chronic, the dose received, the route of exposure, and details of the individual person such as age and health. As an example, lead toxicity affects almost all organs, but the most severely affected is the nervous system [114]. In adults, long-term exposure results in reduced cognitive performance. More severe signs such as learning difficulties and behavioural problems are seen in infants and young children as they are more sensitive during this phase of neurodevelopment [115]. High levels of contamination with lead may also cause kidney damage; chronic exposure may cause anaemia and hypertension, and reduced fertility in males [116]. In pregnant women, high blood lead levels are associated with premature birth or babies with a low birth weight, and this risk is increased in emaciated women [117]. On an individual level, blood sampling is a quick and easy method of assessing circulating levels of lead and can be used to indicate recent or current exposure. However, this does not account for lead stored elsewhere in the body, particularly in bones. X-ray fluorescence can measure whole-body lead in bones, and x-rays may show lead-containing foreign materials [118–120]. Treatment of clinical cases is by using chelating agents, which will reduce blood lead levels, yet neurological effects may remain [121].

On the other hand, at a community level, it may be more important to identify contaminated sites and assess health risks to the general population and thereafter aim to reduce or remove exposure to contaminants such as lead. Thus, monitoring plays a vital role in food safety. Such monitoring has identified contamination of many foods (examples are shown in Table 2). In order to monitor effectively, samples should be analysed from a variety of sources: human samples to detect levels after exposure, diverse foods from the total diet and drinking water sources, and also the environment itself (to identify the source of food contamination). Samples from people frequently include blood, urine, feces, breast milk, hair, and/or semen [122]. Human biomonitoring is notably useful to facilitate risk assessment. A combination of environmental monitoring and biomonitoring may identify risk factors, such as detection of higher levels of cadmium in umbilical cord blood from mothers consuming more than two portions of fish each week [123]. In the case of metals, environmental sampling has shown hotspots of contamination around mining (such as gold, lead, and zinc), electronic waste sites, and industrial areas [110, 124, 125]. Contamination in soils at these sites has been linked to bioaccumulation in agricultural crops and associated increase in human health risk.

Examples of indirect monitoring methods for contaminants in the environment and food include measurement of biomarkers such as proteomics in oysters contaminated with mercury, transcriptome effects in the hepatopancreas of clams, or mutagenicity of seawater in seafood farms associated with PAHs and PCBs [151–153]. High throughput and ultrasensitive screening using nanoparticles has also been utilized for detection of environmental pollutants [154]. Moving forward, testing of chemicals to evaluate potential toxicities before registration and authorised use in the environment may employ tools and concepts such as biomonitoring equivalents and threshold of toxicologic concern, alongside generic and physiologically-based toxicokinetic models [155]. Since 2006, the European Commission has implemented new legislation, called REACH (EC 1907/2006), to identify properties—including toxicities—of chemicals and thus better protect human health and the environment [156]. Other similar legislation exists elsewhere in the world; for example, the Environmental Protection Agency runs a registration process for pesticides to comply with federal laws in the US [157].

Once sources of contaminants have been identified, it is vital to minimize contamination of food. For this purpose, regulations are in place at both national and international levels to restrict contaminated food entering the human food chain. In some cases, legislation exists to assess levels of food contamination. For example, the Marine Strategy Framework Directive in Spain monitors for contaminants in edible tissues of seafood destined for human consumption, assessing levels against established EU standards for food safety [158]. The German Federal Environment Agency monitors both the environment—using the German Environmental Survey (GerES)—and human biomonitoring—using the German Environmental Specimen Bank (ESB) [159]. Amongst others, these have, respectively, been used to detect lead in drinking water and exposure to phthalates and bisphenol A. National monitoring systems may cooperate at an international level. To maintain and improve food safety globally, the Codex Alimentarius contains a set of international food standards, guidelines, and codes of practice [20]. These are based on science from risk assessment bodies or organized by consultations with FAO and WHO. These are voluntary but often form the basis of national legislation.

Food standards and legislation focus on individual food products. To understand the combined risk that someone has from one or many chemicals, a complete dietary risk assessment can be conducted to assess the total potential risk of a typical diet. For example, Zhou et al. assessed the levels of organochlorine pesticides (OCPs) in a total diet from China [160]. The study found that aquatic foods, meats, and cereals were the major foods contributing to contamination of the diet with these chemicals. Multilevel risk assessment can also be used to identify critical points in contamination sources. For example, a study of metals in soil and food in Taiwan identified more than 600 metal-contaminated sites over a period of two decades which could then be targeted for remediation efforts [161].

## 4. Remediation

Once sources of contamination have been identified, it is possible to consider how best to improve food safety through various methods. Methods of remediation vary depending on the type of contaminants present and in which environment. These can be expensive on a large scale. Remediation may focus on reducing contaminants in the environment overall or reducing concentrations in foods specifically.

A common method used to reduce environmental exposure to contaminants is soil remediation. One simple method is to remove contaminated topsoil, which typically contains higher levels of contaminants than subsoil, from agricultural areas [161]. Alternatively, soil turnover and mixing* in situ* may be sufficient to dilute contaminant, such as metals, concentrations to an acceptable level. Thermal treatment or landfill can also be used to remediate a site. Different soil properties can affect contaminant levels. For example, metal (cadmium, mercury, and chromium) accumulation in flowering Chinese cabbage was shown to be controlled by total metal concentrations in soil and available calcium [162]. It is well known that soil science can be used to improve food quality and quantity [163]. It can similarly be used to reduce contamination of crops. The predominant congener of technical DDT,* p,p'*-DDT, is susceptible to microbial metabolism and rarely accumulates in aerobic soils [164]. Long-term gardening has been shown to result in lower levels of PAHs, possibly due to PAH degradation by enhanced microbial activity, and/or dilution [9]. Microbial bioremediation may also be used to reduce levels of metal contamination of soils in an environmentally-friendly manner [165].

Different forms of phytoremediation may be used to either remove contamination from soils or to reduce contamination of plants. If the plants are crops for consumption, reduced uptake is beneficial. One example of phytoremediation is selection of plants to specifically remove contaminants from agricultural land, such as using black nightshade (*Solanum nigrum *L.) for removal of thallium from soil [166]. A study by Yu et al. on cadmium-contaminated agricultural land showed differential accumulation of cadmium in two oilseed rape cultivars [167]. Interestingly, the study also showed increased uptake of cadmium in rice crops planted after the oilseed rape harvest, with contamination of rice higher compared to a crop after a fallow period. Another mechanism of plants which can be used advantageously is that of reduced accumulation of unwanted chemicals in certain cultivars or altered plant hybrids—for example in Chinese kale—and these can be selected to produce safer food [168].

Crop management techniques can affect contamination of plants. Use of slow-release nitrogen fertilizers can reduce cadmium levels in plants such as pak choi, as the plants appear to have stronger tolerance to the metal and a lower efficiency of translocation to edible plant parts compared to those grown using typical fertilizers [169]. Contaminated water used to flood paddy fields is a huge problem in countries that rely on rice crops. Water management—such as drying the paddy field for a period of days between late tillering and young ear differentiation stages—has been shown to reduce cadmium and arsenic levels in rice crops of different rice species [170]. Human health risks from medicinal products contaminating food and water for consumption may be modelled, for example, using pond aquaculture, to identify potential health risks [171].

Exposure to contaminants on foods prepared for consumption can also be reduced by using safer storage alternatives, such as edible films and coatings [172]. Contamination of foods with PAHs from cooking can be greatly reduced by avoiding smoking or open fires but rather replacing them with gas stoves for cooking [77]. Several nongovernmental organizations and charities offer gas stoves to families to help alleviate this source of food contamination, which is a risk particularly for women and children who spend more time at home [173].

## 5. Summary and Conclusions

Attitudes in society towards food safety and contamination are often rooted in tradition and habit. Although consumers select their diet based on social and financial factors, the remit for food safety remains firmly with regulatory bodies. These bodies can monitor for contaminants and enforce legislation. Aspects of food contamination also have political implications. As mentioned above, food safety laws are necessary, with monitoring of food and water contamination, as well as enacting measures to reduce and eliminate exposure to environmental pollutants. Publicity after environmental pollution-related incidents behoves a government to have public health, legal, and ethical frameworks in place in a timely manner. Education of society regarding safer crop cultivation and livestock rearing, selection of a balanced diet, and safer cooking methods should also be encouraged. On a nationwide scale, governments also should endeavour to reduce urban disparities in environmental exposures. Although some contaminants have focal effects, many are transported globally. For this reason, an international stance on food safety is necessary by reducing environmental and food contamination and ensuring trade of safer food products on a global scale.

## Figures and Tables

**Table 1:** Possible human health hazards due to exposure to food contaminants.

**Food contaminants**	**Possible hazards**	**References**
**Metals/metalloids**		

Lead	Complications in the nervous system and red blood cells	[27]
	Reduction in cognitive development and intellectual performance	[28]
	Death among children	[29]

Cadmium	Renal tubular dysfunction, associated with high risk of lung and breast cancer	[30]
	Osteomalacia and osteoporosis	

Arsenic	Associated with dermal, respiratory, nervous, mutagenic, and carcinogenic effects	[31]

Nickel	Associated with dermatotoxicity, lower body weight, and fetotoxicity among pregnant women	[32]

Mercury	Linked to cardiovascular, reproductive, and developmental toxicity, neurotoxicity, nephrotoxicity, immunotoxicity, and carcinogenicity	[33]

**Mycotoxins**		

Aflatoxin	Immunodeficiency	[34]
	Aflatoxicosis	[35]
	Primary hepatocellular carcinoma	[36]
	Liver cirrhosis	[37]

Ochratoxin	Nephropathy	[38]

Deoxynivalenol	Impaired intestinal integrity	[39]
	Impaired gut-associated immune system	

Zearalenone	Hyperestrogenism and reproductive dysfunction	[40]

Fumonisins	Esophageal cancer and birth defects	[41]

**Antimicrobials**		

Tetracyclines	Impaired intestinal flora	[42]

Quinolones	Drug-resistant pathogens	[43]

Macrolides	Hypersensitivity and anaphylactic shock	[44]

Sulfonamides	Kidney damage and nephropathy	[45]

**Polycyclic aromatic hydrocarbons (PAHs)**		

Benzo[a]pyrene	Mutagenicity and carcinogenicity	[46]
	DNA damage and oxidative stress	[47]
	Impaired male fertility	[48]
	Respiratory diseases	[49]
	Cognitive dysfunction among children	[50]

**Pesticides**		

Chlorpyrifos	Neurological symptoms	[51]

DDTs	Neurological symptoms	[52]
	Endocrine disruption	[53]

DDT and other OCPs	Infertility and fetal malformation	[54]

**Dioxins and polychlorinated biphenyls**		

Dioxins and PCBs	Language delay	[55]
	Disturbances in mental and motor development	[56]

PCBs	Neurological disorders	[57]

**Table 2:** Examples of food contamination with different chemicals around the world.

**Food contaminants**	**Foods**	**Country**	**References**
**Metals/metalloids**			

Pb, Hg	Grains and vegetables	China	[58, 62, 67]

Pb, Cd	Livestock organs	Morocco	[59]

Pb	Sheep livers	Spain	[60]

Pb, Cd, Cu, Zn	Agricultural crops	Romania	[61]

Cd	Locally produced foods	Belgium	[64]

Tl	Lettuce and chard	Germany	[65]

Pb, Cd	Soybeans	Argentina	[66]

Cu, Zn, Mn, Fe	Fish	Turkey	[72]

**Mycotoxins**			

Deoxynivalenol, zearalenone, T2 toxin and HT-2 toxin	Wheat, barley, Japanese retail foods	Japan	[126]

Aflatoxin, ochratoxin	Wheat flour	China	[127]

Fumonisins	Maize	South Africa	[128]

Nivalenol	Cereals and cereal products	Tunisia	[129]

Aflatoxins	Ground nut oil	Sudan	[130]

**Antimicrobials**			

Antimicrobials	Pork meat	Madagascar	[131]
	Table eggs	Sudan	[132]
	Milk	Peru	[133]
	Beef	Nigeria	[134]
	Meat	Brazil	[135]

**Polycyclic aromatic hydrocarbons (PAHs)**			

Benzo[a]pyrene	Barbecued foods	Sweden	[136]
Chrysene			

Anthracene	Yogurt	Italy	[137]
Fluoranthene			

19 PAHs	Grains, flour, and bran	Poland	[138]

Total PAHs	Oyster	Japan	[139]

**Pesticides and polychlorinated biphenyls (PCBs)**			

Chlorpyrifos	Catfish	Australia	[140]
	Vegetables	China	[141]
	Food plant	Algeria	[142]

DDTs and other OCPs	Edible offal	Egypt	[143, 144]
	Milk		
	Chicken products	South Africa	[145]
	Milk	Ethiopia	[146]
	Fish	Mozambique	[6]

PCBs and OCPs	Baby foods	Korea	[147]

OCPs and pyrethroids	Honey	Egypt	[148]

PCBs and OCPs	Cereals	Poland	[149]

PCBs and OCPs	Milk, yak muscle and liver	Tibet Plateau	[150]

**Radioactive substances**			

Radioactive substances	Water and food	Japan	[103]

Radionuclides	Seafood	India	[104]

Uranium isotopes	Food	Balkans	[105]

Radioactive substances	Water and food	Switzerland	[106]
